# Synthesis and Nano-Sized Characterization of Bioactive Oregano Essential Oil Molecule-Loaded Small Unilamellar Nanoliposomes with Antifungal Potentialities

**DOI:** 10.3390/molecules26102880

**Published:** 2021-05-13

**Authors:** Katya M. Aguilar-Pérez, Dora I. Medina, Jayanthi Narayanan, Roberto Parra-Saldívar, Hafiz M. N. Iqbal

**Affiliations:** 1 Tecnologico de Monterrey, School of Engineering and Sciences, Atizapan de Zaragoza 52926, Estado de Mexico, Mexico; katyaguilar2904@gmail.com (K.M.A.-P.); dora.medina@tec.mx (D.I.M.); 2 División de Ingeniería en Nanotecnología, Universidad Politécnica del Valle de México, Av. Mexiquense s/n esquina Av. Universidad Politécnica, Col. Villa Esmeralda, Tultitlan 54910, Estado de México, Mexico; jnarayanan@upvm.edu.mx; 3 Tecnologico de Monterrey, School of Engineering and Sciences, Campus Monterrey, Ave. Eugenio Garza Sada 2501, Monterrey 64849, Nuevo Leon, Mexico; r.parra@tec.mx

**Keywords:** nanoliposomes, particle size distribution, oregano essential oil, antifungal activity, mycelial growth inhibition

## Abstract

The development of greener nano-constructs with noteworthy biological activity is of supreme interest, as a robust choice to minimize the extensive use of synthetic drugs. Essential oils (EOs) and their constituents offer medicinal potentialities because of their extensive biological activity, including the inhibition of fungi species. However, their application as natural antifungal agents are limited due to their volatility, low stability, and restricted administration routes. Nanotechnology is receiving particular attention to overcome the drawbacks of EOs such as volatility, degradation, and high sensitivity to environmental/external factors. For the aforementioned reasons, nanoencapsulation of bioactive compounds, for instance, EOs, facilitates protection and controlled-release attributes. Nanoliposomes are bilayer vesicles, at nanoscale, composed of phospholipids, and can encapsulate hydrophilic and hydrophobic compounds. Considering the above critiques, herein, we report the in-house fabrication and nano-size characterization of bioactive oregano essential oil (*Origanum vulgare* L.) (OEO) molecules loaded with small unilamellar vesicles (SUV) nanoliposomes. The study was focused on three main points: (1) multi-compositional fabrication nanoliposomes using a thin film hydration–sonication method; (2) nano-size characterization using various analytical and imaging techniques; and (3) antifungal efficacy of as-developed OEO nanoliposomes against *Trichophyton rubrum* (*T. rubrum*) by performing the mycelial growth inhibition test (MGI). The mean size of the nanoliposomes was around 77.46 ± 0.66 nm and 110.4 ± 0.98 nm, polydispersity index (PdI) of 0.413 ± 0.015, zeta potential values up to −36.94 ± 0.36 mV were obtained by dynamic light scattering (DLS). and spherical morphology was confirmed by scanning electron microscopy (SEM). The presence of OEO into nanoliposomes was displayed by attenuated total reflection Fourier-transform infrared (ATR-FTIR) spectroscopy. Entrapment efficiency values of 79.55 ± 6.9% were achieved for OEO nanoliposomes. In vitro antifungal activity of nanoliposomes tested against *T. rubrum* strains revealed that OEO nanoliposomes exhibited the highest MGI, 81.66 ± 0.86%, at a concentration of 1.5 µL/mL compared to the rest of the formulations. In summary, this work showed that bioactive OEO molecules with loaded nanoliposomes could be used as natural antifungal agents for therapeutical purposes against *T. rubrum*.

## 1. Introduction

The demand for bioactive molecules or bioactive agent-loaded nano-constructs with desirable properties has increased in the past few years [1], as an alternative and robust choice to minimize the extensive use of synthetic drugs. Therefore, carriers of bioactive molecules or drug-loaded constructs must be able to retain their contents until reaching target sites in the body [2]. A functional approach to perform efficient delivery with target specificity and controlled release is the use of nanomaterials (e.g., nanofibers, nanogels, micelles, nanoparticles, nanoliposomes, etc.). Among these nanomaterials, nanoliposomes represent the most used phospholipid-based nanocarrier for drug delivery applications [3]. Nanoliposomes are bilayer structures that maintain their nanometric size within the range of 20 to 150 nm, during storage and applications. Additionally, their lipid and aqueous composition allow the entrapment of hydrophobic and hydrophilic agents, either individually or simultaneously [4]. According to the number of lamellae, size, and preparation method, phospholipid vesicles can be classified in several groups [5]. However, classifications by the number of layers and size are most commonly used [6], such as, SUV: small unilamellar vesicles (20–50 nm); LUV: large unilamellar vesicles (>560 nm); MLV: multilamellar vesicles (170–5000 nm); OLV: oligolamellar vesicles; MUV: medium unilamellar vesicles (unilamellar vesicles; >100 nm); GUV: giant unilamellar vesicles (cell size vesicles with diameters >1 mm). In unilamellar structures, the vesicle has a single phospholipid bilayer sphere enclosing the aqueous solution. In multilamellar liposomes, vesicles have an onion structure. Classically, several unilamellar vesicles will form on the inside of those with a smaller size, making a multilamellar structure of concentric phospholipid spheres separated by layers of water [7]. Overall, nanoliposomes have an affinity to the biological membrane, which facilitates their cellular absorption. In addition, they are non-toxic, biodegradable, biocompatible, and can be prepared using natural ingredients (e.g., egg yolk, soybean lecithin, sunflower oil, etc.) [8].

Dermatophytosis is one of the most common infections, affecting 20 to 25% of the world population. Among the most prevalent causative agents related to this infection, *Trichophyton rubrum* species have been reported as responsible for infecting keratinized tissues such as nails, hairs, and skin [9]. Despite the great efforts in developing antifungal treatments, there is still a current necessity for treatments with high efficacy and low toxicity. Moreover, the opportunity of using plant-based materials for medical purposes is gaining attention among research groups [10]. Essential oils (EOs) are complex mixtures of natural compounds obtained mainly from herbs and spices [11]. EOs have been used as an alternative therapeutic system to conventional drugs in view of their antinociceptive effects, antioxidant, antibacterial, and antifungal activity [12]. For instance, Oregano essential oil (OEO) is extracted from Oregano (*Origanum vulgare* L.), which has been recognized as one of the most important medicinal plants highly reputed as an efficient remedy for infectious diseases. OEO is mainly composed of carvacrol (57.4–69.6%), thymol (30.3–42.8%), cymene (17.7–51.3%), and terpinene (2.63–6.15%) [13]. These bioactive compounds have strong antibacterial, antifungal, antiparasitic, cytotoxic, and antioxidant activity [14,15,16,17,18]. However, the high volatility, sensitivity, and relatively low stability of EOs may minimize their performance when intended for use as pharmaceutical agents [15].

In this regard, incorporation of EOs into nanoliposomes holds a great strategy to improve bioavailability, minimize toxicity, and prevent the degradation of EOs for therapeutical proposes [13,14,15]. Thus, the combination of essential oils with engineered materials has made it possible to enhance EO features by protecting them from external factors, therefore avoiding degradation, oxidation, and reducing their volatility [17]. The purpose of this work was to fabricate and characterize OEO-loaded nanoliposomes. Additionally, a comparison study between OEO in bulk and encapsulated into nanoliposomes was performed by testing the mycelial growth inhibition (MGI) to measure their effectiveness against fungal strains of *Trichophyton rubrum.*

## 2. Results and Discussion

### 2.1. Size Distribution of OEO Nanoliposomes

Particle size is one of the most influencing factors guiding the behavior of nanomaterials, stability, release profiles, biological distribution, and cellular uptake. It has been stated that decreasing the size of nanoliposomes promotes their permeation through intercellular paths [19]. Additionally, the polydispersity index (PdI) of lipid-based nanovesicles may affect their stability and is considered a key factor in particle size distribution. PdI values can range from 0 to 1.0 for an adequately monodisperse and polydisperse nanovesicle, respectively [20].

**Figure 1:**
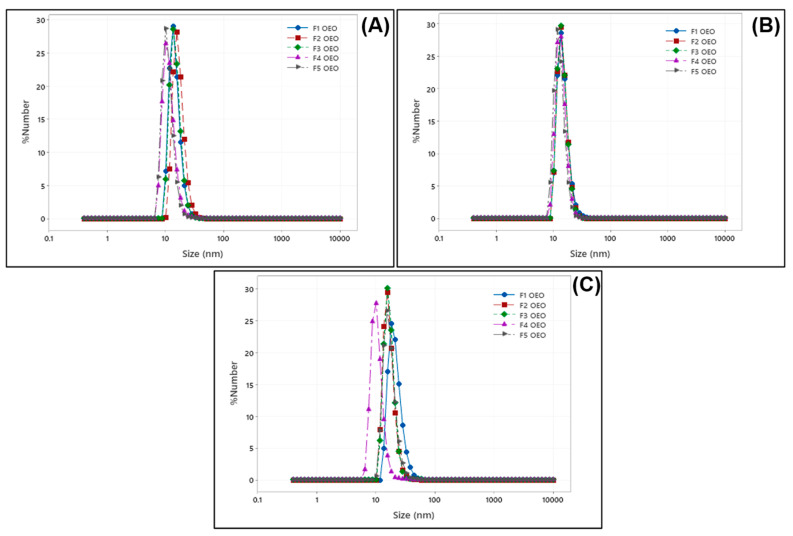
Average particle size distribution of OEO nanoliposomes (**A**) after 1 day of preparation, (**B**), after 1 week of preparation, and (**C**) after 1 month of preparation.

For the case of OEO-loaded nanoliposomes, the smallest particle size was obtained at higher concentrations of OEO, labeled as F1 OEO (79.63 ± 0.92 nm at 6.2 mM). During the first measurement and after 1 week of storage, F1 OEO kept the smallest particle size, as shown in Table 1. However, after 1 month, its particle size increased and was the largest particle size compared to the rest of the formulations. Changes in the particle size of nanoliposomes are attributed to the small average particle size. The smaller the particle size, the higher the surface area, and the strong attractive interaction between particles derived the aggregation agglomeration [21]. For the rest of the formulations, particle size values ranged between 81.83 ± 1.06 nm and 87.30 ± 0.60 nm, as shown in Figure 1A–C. The nanoliposome formulations were bigger in size during the first measurement with respect to the control sample. However, after 1 week and 1 month, the control vesicle (empty nanoliposome) exhibited the highest particle size with respect to the rest of the formulations loaded with OEO. Lipid composition and encapsulated compounds affect the size of nanoliposomes. In this regard, the bulk of nanoliposomes would be larger in the case of the addition of compounds such as cholesterol [22].

The main components of OEO are terpenes, generally mono and sesquiterpenes, with carvacrol, thymol, γ-terpinene, and p-cymene some of the main constituents [23]. It has been reported that small terpenes can decrease the size of lipid nanocarriers by compelling the phosphatidylcholine structure to increase its surface curvature [24]. For OEO nanoliposomes, PdI values were lower than 0.5 in all cases, indicating a monodisperse system. Several literature reports related to the encapsulation of EOs into nanoliposomes, for instance, pistachio oil (100–250 nm) and PdI (0.1–0.5) [25], rosemary oil (79–142.5 nm) and PdI (0.893–1.000) [26], and Zataria multiflora oil (97.8–210 nm) and PdI (0.172–0.424) [27] are close to the obtained results in this work. In this regard, the size of OEO nanoliposomes meets the conditions specified for pharmaceutical and other delivery applications.

### 2.2. Zeta Potential Values of OEO Nanoliposomes

Zeta potential measurement is a common practice to determine the surface charge of colloidal systems, repulsion forces between them, and their physical stability [28]. Hence, the stability of nanoliposomes directly increases the increase in the repulsion of particles by either the electrostatic or steric repulsion of vesicles [20]. Acceptable zeta potential values to consider a system as stable range from lower than −30 mV to higher +30 mV [29] and are considered suitable for the colloidal stability of nanoliposomes [30]. Nanoliposome formulations loaded with OEO presented zeta potential values in the range of −36.94 ± 0.36 mV, higher compared to the control samples, which exhibited values from −15.54 ± 1.49 mV to −8.66 ± 1.43 mV (Table 2). The sign and magnitude of zeta potential was determined by the net charge accumulated on the nanoliposome surface. OEO nanoliposomes exhibited negative zeta potential values. These results may be attributed, on one hand, to the choline head present in the phosphatidylcholine group of soybean lecithin [31], which is zwitterionic. On the other hand, according to Makino’s model [32,33], its polar head can reorient depending on the temperature ionic strength. At a low ionic strength, the choline groups are located below the phosphate group (negative zeta potential), whereas at a high ionic strength, the situation is reversed. Moreover, a 1 X PBS solution at pH 7.4 was used during the hydration process which involved anionic species, and their dissociation equilibria involved multivalent anions which may lead to negative zeta potential values [34,35].

**Figure 2:**
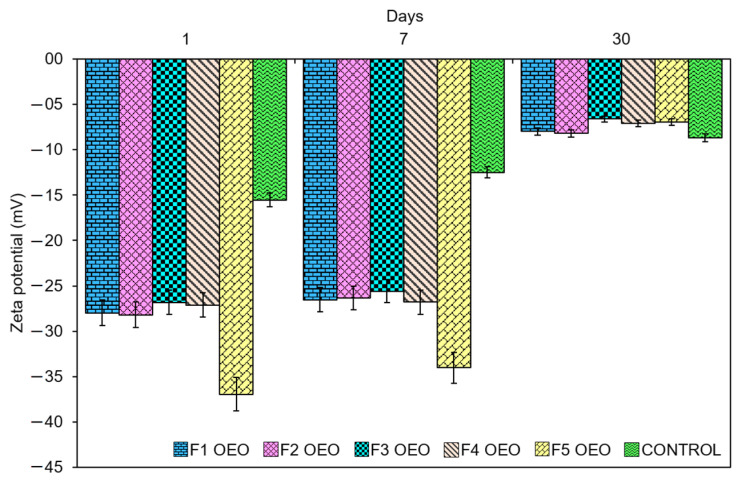
Zeta potential values of OEO nanoliposomes and control sample after 1 day, 1 week, and 1 month of preparation.

It was observed that zeta potential values were higher at lower OEO concentrations in all the measurements performed. However, all the formulations reached lower zeta potential values after 1 month of storage, such as the case of formulation F3 OEO (−6.62 ± 0.75 mV). A decrease in zeta potential is associated to small particle size, large surface area, and free energy of nanoparticles. If zeta potential values are low, attraction overcomes repulsion in order to minimize the energy, and the mixture forms aggregates [36]. Zeta potential values of OEO nanoliposomes and control samples over time are presented in Figure 2. This phenomenon has previously been reported by [37] and can be attributed to the sonication energy introduced into the system during preparation, resulting in coalescence or aggregation during storage. Therefore, there was not a significant contribution of Tween-80^®^ in the stability of nanoliposomes under storage. However, further studies could be performed in the future, varying the amount of surfactant at different concentrations of OEO.

### 2.3. Scanning Electron Microscopy (SEM) Analysis

**Figure 3:**
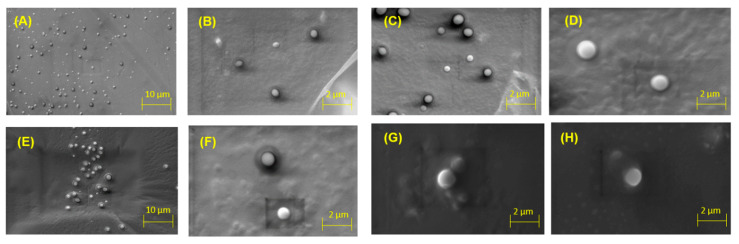
Scanning electron microscope (SEM) images of OEO nanoliposomes at different magnifications (top line): 1.00 K (**A**), 2.00 K (**B**), 4.00 K (**C**) and 6.00 K (**D**), and control nanoliposomes (bottom line): 1.00 K (**E**), 2.00 K (**F**), 4.00 K (**G**) and 6.00 K (**H**).

Morphology plays an essential role in the performance of nanoliposomes. For instance, in medical applications, the morphology of nanoliposomes influences their surface ratio and might affect the drug distribution in the blood, circulation time, and targeting efficiency [38,39]. OEO nanoliposomes were observed under 1.0 K, 2.0 K, 4.0 K and 6.0 K magnifications to confirm their spherical shape and distribution. As shown in Figure 3, spherical vesicles and closed-continuous structures of the nanoliposome samples were observed.

### 2.4. ATR-FTIR Studies of OEO Nanoliposomes

**Figure 4:**
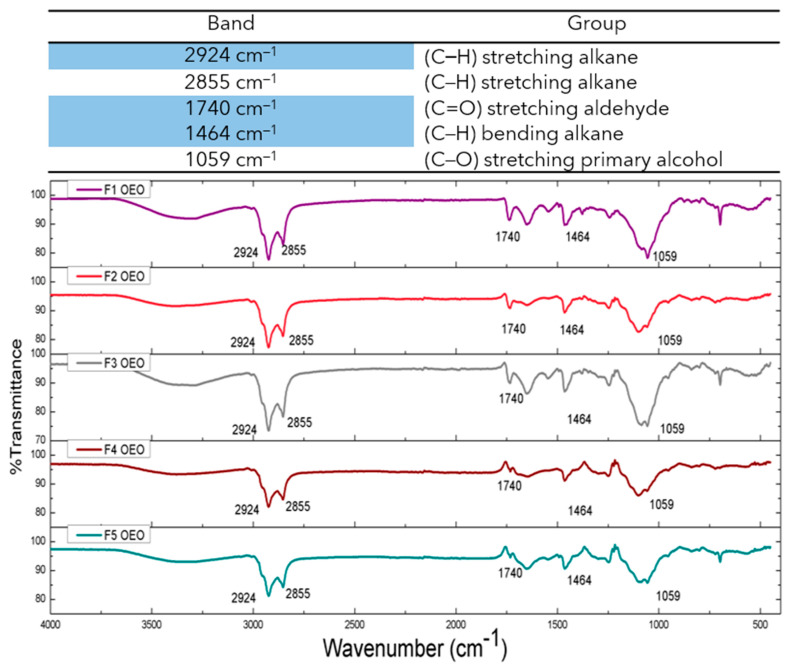
Infrared spectra of five formulations of OEO nanoliposomes at different concentrations (6.2, 4.9, 3.7, 2.5 and 1.2 mM).

**Figure 5:**
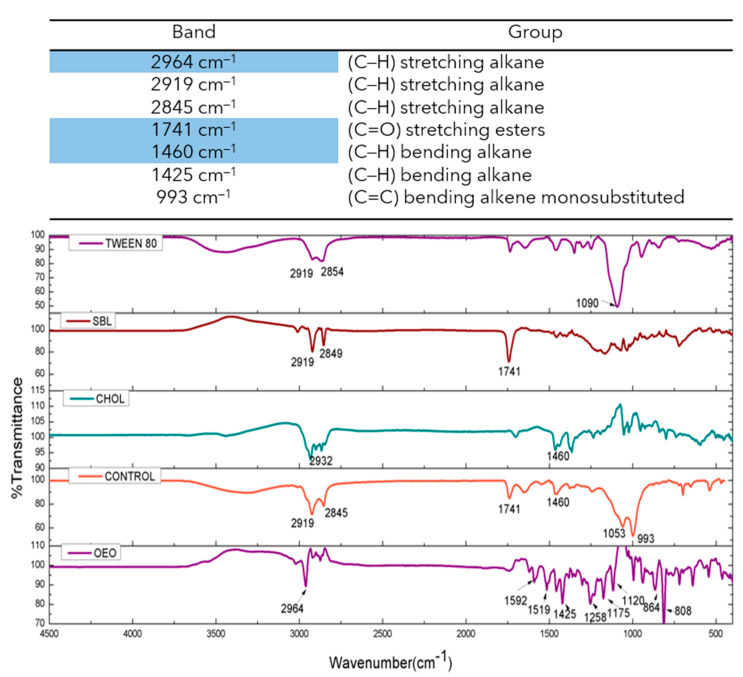
Infrared spectra of the main ingredients of nanoliposomes (SBL, Chol, Tween-80^®^, OEO and the empty nanoliposome referred to as the control).

The FTIR spectra of OEO, empty nanoliposomes, OEO-loaded nanoliposomes, and phospholipid constituents were obtained and are presented in Figure 4 and Figure 5. Nanoliposome samples loaded with OEO presented vibrations at 2924 cm^−1^ (C–H) stretching alkane, 2855 cm^−1^ (C–H) stretching alkane, 1740 cm^−1^ (C=O) stretching aldehyde, 1464 cm^−1^ (C–H) bending alkane, and 1059 cm^−1^ (C–O) stretching primary alcohol. The FTIR spectrum of pure OEO exhibited a high number of vibrations related to the volatile compounds, and the bands at 2964 cm^−1^ (C–H) stretching alkane and 1425 cm^−1^ (C–H) bending alkane, this latter related to the methyl group, showed a shift in the nanoliposome formulation containing OEO, which can be attributed to the stretching vibration of the C–H groups and the possible overlapping between bands due to the change in the strength of molecular interactions [31,40]. The empty nanoliposome revealed vibrations at 2919 cm^−1^ (C–H), and 2845 cm^−1^ (C–H) related to stretching alkanes, 1741 cm^−1^ (C=O) stretching esters, 1460 cm^−1^ (C–H) bending alkane related to methylene groups, 1053 cm^−1^ (C–O) stretching primary alcohol, and 993 cm^−1^ (C=C) bending alkene monosubstituted, indicating an increase in stability of alkenes due to the substitution of hydrogen for carbon [34]. These results showed the appropriate incorporation of OEO into the phospholipid matrix.

### 2.5. Entrapment Efficiency (EE%)

**Figure 6:**
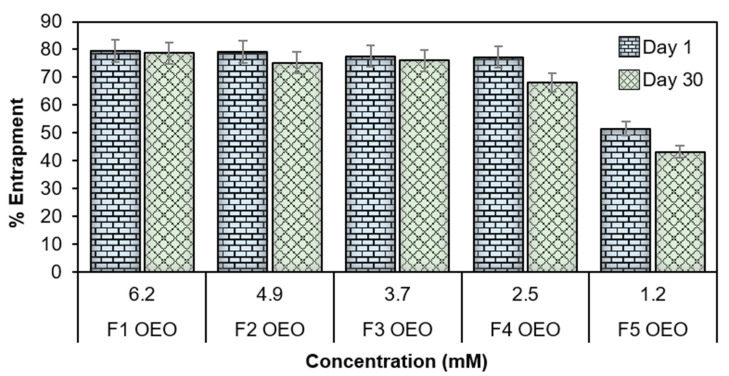
Entrapment efficiency percentage (%EE) of OEO nanoliposomes 1 day and 30 days after preparation.

Entrapment efficiency percentage (EE%) is an important index to determine the capability of nanosystems in preserving and stabilizing the encapsulated compounds [41]. In this study, the EE% test revealed that the set of OEO-loaded nanoliposomes reached values up to 79.55 ± 6.9% (see Table 3). It can be observed that the EE% had slight variance after 30 days, and the percentage values were affected directly by the concentration of OEO, as presented in Figure 6. The EE% values are summarized in Table 3 for the set of loaded and empty nanoliposome formulations tested 1 and 30 days after the synthesis process. It was observed that the highest EE% values were obtained at higher concentrations of OEO, this may be attributed firstly due to the incorporation of cholesterol during preparation which decreases its permeability and favors encapsulation by decreasing the flexibility of the surrounding lipid chains [4,6,42]. On the other hand, some studies have evaluated the EE% of EO components [43], reporting that better incorporation of EOs into nanoliposomes membranes is given by the interaction of the hydroxyl groups present in EOs with the membrane components of nanoliposomes (e.g., cholesterol, soybean lecithin, etc.); in this case, the hydroxyl groups act as hydrogen bond donors, and the phosphate head groups of phospholipids act as hydrogen bond acceptors [44].

### 2.6. In Vitro Antifungal Activity of Nanoliposomes—Mycelial Growth Test

**Figure 7:**
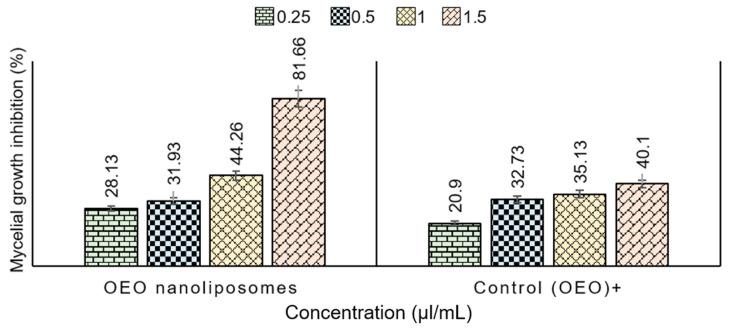
Evaluation of antifungal activity of EO nanoliposomes (OEO) against *T. rubrum* strains. The analysis was performed at four different concentrations for all the samples and the %MGI was calculated according to the formula described in the experimental section.

In this study, OEO oils loaded into nanoliposomes were tested against fungal strains of *T. rubrum* by using the agar dilution method [10]. With respect to the prepared nanoliposome formulations, it was found that OEO nanoliposomes reached %MGI values in the range of 81.66 ± 0.86% at 1.5 µL/mL (Table 4). Additionally, pure EO (OEO) in bulk was used as a positive control to observe if there was an improvement in antifungal activity after the encapsulation process. The highest %MGI of OEO in bulk was 40.1 ± 2.16% at 1.5 µL/mL concentration. This is shown in Figure 7, thus confirming that %MGI values of OEO nanoliposomes were higher than their respective positive controls at all concentrations.

These results state the effectiveness of encapsulation of EOs into nanoliposomes to increase the antifungal activity against *T. rubrum* strains. Remarkably, it is interesting that even at the lowest concentrations of EO (0.25 µL/mL), the inhibition (28.13 ± 1.72%) values were higher than the control nanoliposomes (20.9 ± 1.51%) encapsulated into nanoliposomes, and there was an increase in %MGI in all the formulations compared to the EOs in bulk.

## 3. Materials and Methods

### 3.1. Materials/Chemicals/Reagents

**Figure 8:**
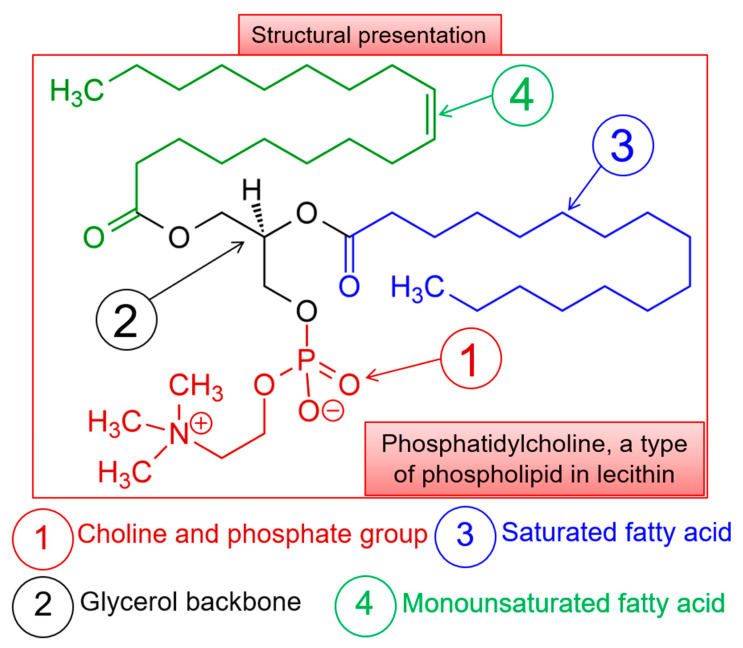
Structural representation of SBL phosphatidylcholine along with its structural backbone entities.

Soybean lecithin (SBL) phosphatidylcholine was purchased from a local market (Nuevo León, México). Figure 8 illustrates the structural representation of SBL phosphatidylcholine along with its structural backbone entities. Cholesterol (Cho) was obtained from Sigma Aldrich (Darmsdat, Germany). Oregano essential oil (OEO) was purchased from PACALI (Nuevo León, México). Tween 80^®^ was obtained from Sigma Aldrich (Darmsdat, Germany). Chloroform was purchased from J.T.Baker™ (Bridgewater, NJ, USA) and phosphate-buffered saline (PBS) solution was obtained from ThermoFisher (Waltham, MA, USA).

### 3.2. Preparation of Nanoliposomes in the Presence/Absence of OEO

**Figure 9:**
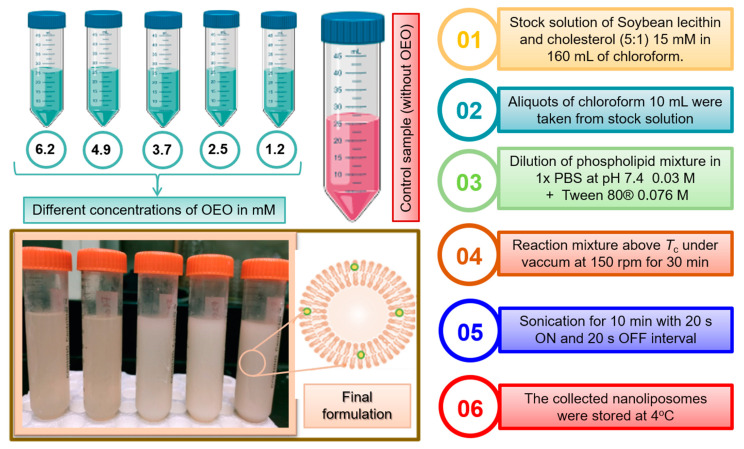
Schematic representation of OEO nanoliposome formulations at different concentrations (6.2, 4.9, 3.7, 2.5 and 1.2 mM) and empty nanoliposomes referred to as controls. Final milky solution at the bottom-left of the picture represents OEO nanoliposomes.

The thin film hydration–sonication method was used to carry out the synthesis of nanoliposomes. Briefly, a stock solution of 160 mL at 15 mM was prepared using the lipid components, i.e., SBL and Chol in a 5:1 ratio and dissolved in chloroform. Aliquots of 10 mL were taken and dissolved with the essential oil at different concentrations: 6.2, 4.9, 3.7, 2.5 and 1.2 mM (Figure 9). The solvent was removed using a rotary evaporator above the 60 °C transition temperature of the lipid at 150 rpm, and a high vacuum, which resulted in a uniform thin lipid membrane on the vessel wall. Traces of organic solvents were removed under vacuum for 1 h, and then by storage of the samples for 24 h in a desiccator. The deposited lipid membrane was hydrated with 20 mL of PBS solution, pH 7.4, containing 1% Tween 80, and by agitation for 30 min at 60 °C. Simultaneously, sonication of the preparation was performed by using a sonicator probe with 60% amplitude of sonication for 10 min with 20 s ON and 20 s OFF intervals to avoid heating and to form small unilamellar vesicles. Finally, the liposomal dispersions were stored at 4 °C for 1 month.

### 3.3. Characterization of Nanoliposomes

Characterization of nanoliposomes was performed by measuring the vesicle size and surface charge using the dynamic light scattering (DLS) technique. The morphological characterization was analyzed by scanning electron microscopy (SEM). The chemical interactions between the phenolic compounds from essential oils and nanoliposomes were evaluated by attenuated total reflection Fourier-transform infrared spectroscopy (ATR-FTIR). Afterwards, the evaluation of nanoliposomes’ in vitro antifungal activity was assessed against fungal strains of *Trichophyton rubrum*.

#### 3.3.1. Dynamic Light Scattering (DLS)

Average particle size, zeta potential, and polydispersity index were measured by integrated dynamic light scattering with a Zetasizer Nano ZS (Malvern Panalytical Instruments, Malvern, WR14 1XZ, United Kingdom) at 25 °C for a duration of 60 s. Data were analyzed using MALVERN software. To avoid multiple scattering, 200 µL of the samples was diluted (5:2) with a PBS solution and placed in a disposable polystyrene cuvette DTS0012. For measurements of the zeta potential, the samples were correspondingly diluted with PBS solution and transferred to a Universal Dip Cell (ZEN 1002). The measurements were performed the 1st day, 1st week, and 1st month after preparation by triplicate for each sample.

#### 3.3.2. Scanning Electron Microscopy (SEM) Analysis

The morphology of the nanoliposomes was analyzed by scanning electron microscopy (ZEISS EVO^®^ MA 25, Ostalbkreis, Baden-Württemberg, Germany) at EHT: 15.00 kV. To confirm the spherical shape of the prepared nanoliposomes, samples containing OEO and empty nanoliposomes were pre-frozen at −80 °C for 24 h and then lyophilized under vacuum. Finally, the samples were coated with gold in a sputter coater under an Ar atmosphere (50 Pa) at 50 mA for 50 s.

#### 3.3.3. Attenuated Total Reflection Fourier-Transform Infrared Spectroscopy (ATR-FTIR)

Attenuated total reflection Fourier-transform infrared spectroscopy (ATR-FTIR) was performed using a spectrophotometer (Frontier IR Single-Range Systems Perkin Elmer, UK) equipment. The formulations were collected by centrifuge at 15,200 rpm for 30 min at 4 °C and washed 3 times with double-distilled water to remove the salts and then lyophilized overnight. The measurements were performed at wavenumber: 4000 to 400 cm^−1^, 16 scans; resolution: 2 cm^−1^; speed: 0.50 cm/s; and force gauge: 100. SBL, Chol and Tween 80^®^ were also analyzed to better understand the vibrations of each compound separately and confirm the incorporation of OEO into nanoliposomes.

#### 3.3.4. Determination of Entrapment Efficiency

The %EE was calculated after the ultracentrifugation of the samples. Aliquots of 33 mL from nanoliposome solutions were taken and placed in a falcon tube. Then, the samples were ultracentrifuged (Optima XE 100, Beckman Coulter, Indianapolis, IN, USA) with a Rotor SW32 TI at 174,000× *g* for 3 h at 4 °C. The unencapsulated portion was accumulated in the bottom of the falcon. The supernatant was collected, and Equation (1) was used to calculate the total amount of entrapped essential oil (OEO). The concentration of unentrapped EO was measured spectrophotometrically with UV–vis (Perkin Elmer, model Lambda 365, Shelton, Connecticut, USA) by measuring the UV absorbance at λ max 298 nm.

(1) $$EE\left(\%\right)=\frac{Total\;amount\;of\;EO-Unentrapped\;EO}{Total\;amount\;of\;EO}\times 100\%$$

### 3.4. Isolation of Dermatophyte Fungi

A fungal strain provided by the Department of Biological Sciences of Universidad Autonoma de Nuevo Leon was used to isolate and reseed *Trichophyton rubrum* species. Briefly, 5.85 g potato dextrose agar (PDA) was diluted in 150 mL of double-distilled water under magnetic stirring. The sample was poured into a flask and sterilized at 121 °C for 1 h and 45 min in the autoclave. Subsequently, the flask was transferred to the laminar flow hood previously sprayed with ethanol solution at 70%. The flask with the PDA solution and a set of Petri dishes were kept in the laminar flow hood under UV light for 15 min. The Petri dishes were filled up to three-quarters of their capacity with the medium and placed under UV light to avoid contamination during agar solidification for 15 min, and then were incubated at 29 °C for 24 h to confirm the inexistence of pollution in the medium. Finally, two sections of the fungal strain were transferred to the medium and incubated at 29 °C for 1 week.

#### In Vitro Antifungal Activity of Nanoliposomes—Mycelial Growth Test

Contact effects of EOs with loaded nanoliposomes and unloaded nanoliposomes on the mycelial growth of *T. rubrum* were assessed in vitro using the agar dilution method [18]. The nanoliposome solutions and control samples were dispersed as an emulsion in sterile PDA containing 1% (*v/v*) Tween-80^®^ immediately before pouring it into glass Petri dishes (8 cm diameter) at a temperature of 40–55°C. The dilutions tested were 0.25, 0.5, 1.0 and 1.5 μL/mL. Control plates received the same quantity of Tween-80^®^ mixed with PDA. A 5 mm diameter disc containing *T. rubrum* was taken from the edge of 7-day-old fungal culture and placed in the center of each plate. After incubation at 25 °C for 3 days, treatment efficacy was determined by measuring the average of two perpendicular diameters through each colony. The percentage inhibition of MGI was calculated according to Equation (2) from mean values of colony diameter in treated and control Petri dishes.

MGI % = [(*dc* − *dt*)/*dc*] × 100 (2)

where *dc* (cm) is equivalent to the mean colony diameter for the control sets, and *dt* (cm) is equal to the colony diameter for the treatment sets. Each analysis was performed in triplicate.

### 3.5. Statistical Analysis

For statistical analysis, Minitab 19 software (2020 Minitab, LLC., State College, PA, USA) was used by performing one-way analysis of variance (ANOVA). The datasets were evaluated at least in triplicate, and *p*-values < 0.05 were considered as statistically significant.

## 4. Concluding Remarks and Outlook

OEO nanoliposomes were prepared by using a thin-film hydration–sonication method. This method enabled obtaining nano-sized liposomes at five different concentrations (6.2, 4.9, 3.7, 2.5 and 1.2 mM). Size and morphology were analyzed by DLS and SEM imaging to confirm that the diameters of the prepared vesicles were within the nanometric scale (77.46 ± 0.66 nm) after 1 day, 1 week and 1 month of preparation; OEO nanoliposomes also exhibited spherical morphology. Entrapment efficiency was evaluated after 1 day and 30 days of preparation by spectroscopic analysis. EE% was found to be affected by EO concentrations and the highest values were 79.55 ± 6.9% and 78.7 ± 5.4%, respectively, at a concentration of 6.2 mM OEO. Therefore, stability of OEO nanoliposomes under storage was evaluated 1 day, 1 week and 1 month following the synthesis, with zeta potential measurements reaching values around −36.94 ± 0.36 mV throughout. It was clear that EOs influenced the size of the nanoliposome; at higher concentrations, there was an increase in the particle size, an effect which might be due to the interaction between EOs as hydrophobic agents with the acyl chains of lecithin, which is responsible for altering the acyl chain order, and the effect was more pronounced when the EO concentration was increased. Moreover, the surface charge and stability of the vesicles were affected by the phospholipids and excipients utilized for the fabrication process. After one month of storage, a significant degree of instability was observed, as demonstrated by zeta potential values (approximately −6.97 ± 0.61 mV), PdI, and particle size, not only in the OEO-loaded nanoliposomes, but also in the empty vesicles taken as controls. These results suggest that there was no significant contribution of the surfactant utilized during the synthesis process (Tween-80^®^) to stabilize the system during long periods. Moreover, the ATR-FTIR studies confirmed the presence of OEO bands in the nanoliposome formulations. Additionally, OEO nanoliposomes’ antifungal activity demonstrated the capability to inhibit mycelial growth at lower concentrations compared to the pure EOs against fungi strains of *T. rubrum.* Finally, it can be concluded that nanoliposomes are suitable for potential applications as antifungal delivery agents. Nevertheless, further stability studies are necessary to analyze the optimal formulation to prevent agglomeration during storage for longer periods.

## Figures and Tables

**Table 1:** Average particle size and PdI of OEO nanoliposomes and control sample at different concentrations after 1 day, 1 week, and 1 month of storage at 4 °C.

Sample Name	OEO Concentration (mM)	Size 1st Day (nm)	PdI 1st Day	Size 1st Week (nm)	PdI 1st Week	Size 1st Month (nm)	PdI 1st Month
F1 OEO	6.2	79.63 ± 0.92	0.413 ± 0.015	77.46 ± 0.66	0.396 ± 0.003	110.4 ± 0.98	0.479 ± 0.049
F2 OEO	4.9	87.30 ± 0.60	0.311 ± 0.008	88.48 ± 1.95	0.338 ± 0.041	92.27 ± 1.58	0.461 ± 0.004
F3 OEO	3.7	81.36 ± 0.75	0.302 ± 0.010	78.30 ± 0.64	0.419 ± 0.005	100.19 ± 0.56	0.490 ± 0.003
F4 OEO	2.5	83.14 ± 2.38	0.402 ± 0.071	81.09 ± 0.55	0.434 ± 0.003	98.51 ± 0.45	0.504 ± 0.007
F5 OEO	1.2	81.83 ± 1.06	0.434 ± 0.930	90.33 ± 0.51	0.350 ± 0.043	101.06 ± 0.76	0.484 ± 0.007
Control	-	32.43 ± 1.47	0.250 ± 0.002	105.10 ± 1.65	0.292 ± 0.007	110.83 ± 0.83	0.464 ± 0.003

Data are expressed as mean values ± standard deviation. Abbreviations: OEO, oregano essential oil; PdI, polydispersity index.

**Table 2:** Zeta potential values of OEO nanoliposomes and control samples at different concentrations after 1 day, 1 week, and 1 month of storage at 4 °C.

Sample Name	OEO Concentration (mM)	Zeta Potential 1st Day (mV)	Zeta Potential 1st Week (mV)	Zeta Potential 1st Month (mV)
F1 OEO	6.2	−27.99 ± 1.15	−26.54 ± 0.36	−7.98 ± 0.93
F2 OEO	4.9	−28.20 ± 0.78	−26.32 ± 0.47	−8.22 ± 1.79
F3 OEO	3.7	−26.82 ± 0.37	−25.59 ± 0.53	−6.62 ± 0.75
F4 OEO	2.5	−27.11 ± 0.52	−26.80 ± 0.44	−7.13 ± 1.07
F5 OEO	1.2	−36.94 ± 0.36	−34.03 ± 0.20	−6.97 ± 0.61
Control	-	−27.54 ± 1.49	−20.5 ± 0.7	−8.66 ± 1.43

Data are expressed as mean values ± standard deviation.

**Table 3:** Entrapment efficiency percentage (EE%) of OEO at different concentrations after 1 day and 1 month of storage at 4 °C. Data are expressed as mean values ± standard deviation.

Sample Name	OEO Concentration (mM)	Day 1	Day 30
F1 OEO	6.2	79.55 ± 6.9	78.7 ± 5.4
F2 OEO	4.9	79.10 ± 5.3	75.3 ± 3.9
F3 OEO	3.7	77.60 ± 4.3	76.1 ± 2.7
F4 OEO	2.5	77.29 ± 2.4	68.09 ± 1.9
F5 OEO	1.2	51.50 ± 1.3	43.2 ± 2.4

**Table 4:** Mycelial growth inhibition (MGI) of OEO nanoliposomes. Pure OEO was taken as a positive control and empty nanoliposomes were taken as a negative control. Data are expressed as mean values ± standard deviation.

OEO Concentration (µL/mL)	MGI (%) OEO Nanoliposomes	MGI (%) Control (OEO)
0.25	28.13 ± 1.72	20.9 ± 1.51
0.5	31.93 ± 1.55	32.73 ± 2.28
1	44.26 ± 3.95	35.13 ± 3.41
1.5	81.66 ± 0.86	40.1 ± 2.16

## Data Availability

All data, belongs to this work, is given and presented herein.

## Sample Availability

Not available.
